# Intracellular Localization, Interactions and Functions of Capsicum Chlorosis Virus Proteins

**DOI:** 10.3389/fmicb.2017.00612

**Published:** 2017-04-11

**Authors:** Shirani M. K. Widana Gamage, Ralf G. Dietzgen

**Affiliations:** Queensland Alliance for Agriculture and Food Innovation, The University of Queensland, St LuciaQLD, Australia

**Keywords:** tospovirus, capsicum chlorosis virus, protein intracellular localization, bimolecular fluorescence complementation, cell-to-cell movement, RNA silencing suppression, immunofluorescence, protoplasts

## Abstract

Tospoviruses are among the most devastating viruses of horticultural and field crops. Capsicum chlorosis virus (CaCV) has emerged as an important pathogen of capsicum and tomato in Australia and South-east Asia. Present knowledge about CaCV protein functions in host cells is lacking. We determined intracellular localization and interactions of CaCV proteins by live plant cell imaging to gain insight into the associations of viral proteins during infection. Proteins were transiently expressed as fusions to autofluorescent proteins in leaf epidermal cells of *Nicotiana benthamiana* and capsicum. All viral proteins localized at least partially in the cell periphery suggestive of cytoplasmic replication and assembly of CaCV. Nucleocapsid (N) and non-structural movement (NSm) proteins localized exclusively in the cell periphery, while non-structural suppressor of silencing (NSs) protein and Gc and Gn glycoproteins accumulated in both the cell periphery and the nucleus. Nuclear localization of CaCV Gn and NSs is unique among tospoviruses. We validated nuclear localization of NSs by immunofluorescence in protoplasts. Bimolecular fluorescence complementation showed self-interactions of CaCV N, NSs and NSm, and heterotypic interactions of N with NSs and Gn. All interactions occurred in the cytoplasm, except NSs self-interaction was exclusively nuclear. Interactions of a tospoviral NSs protein with itself and with N had not been reported previously. Functionally, CaCV NSs showed strong local and systemic RNA silencing suppressor activity and appears to delay short-distance spread of silencing signal. Cell-to-cell movement activity of NSm was demonstrated by *trans*-complementation of a movement-defective tobamovirus replicon. CaCV NSm localized at plasmodesmata and its transient expression led to the formation of tubular structures that protruded from protoplasts. The D_155_ residue in the 30K-like movement protein-specific LxD/N_50-70_G motif of NSm was critical for plasmodesmata localization and movement activity. Compared to other tospoviruses, CaCV proteins have both conserved and unique properties in terms of *in planta* localization, interactions and protein functions which will effect viral multiplication and movement in host plants.

## Introduction

Capsicum chlorosis virus was first discovered in capsicum (*Capsicum annuum* L.) and tomato (*Solanum lycopersicum* L.) crops in Queensland, Australia in 1999 (McMichael et al., 2002). Subsequently, CaCV was recorded in China, India, Taiwan, and Thailand from capsicum, groundnut and tomato crops and ornamental plants (Knierim et al., 2006; Chen C. et al., 2007; Chen K. et al., 2007; Kunkalikar et al., 2007). Due to a lack of natural resistance in commercial cultivars and inefficient control of transmitting thrips vectors, CaCV is of growing concern to horticultural industries.

Taxonomically, CaCV is considered as a tentative species in the genus *Tospovirus*, the only genus in the family *Bunyaviridae* representing plant-infecting viruses (Plyusnin et al., 2011). The genome of CaCV has the typical tospovirus genome structure consisting of three linear RNA segments with negative or ambisense coding polarity (Knierim et al., 2006; Zheng et al., 2011; Widana Gamage et al., 2015). The RNA segments are named as large (L), medium (M), and small (S) based on size. L RNA contains a single open reading frame (ORF), which codes for an RNA-dependent RNA polymerase (RdRP) (de Haan et al., 1991). M RNA contains two ORFs separated by an intergenic region (IGR), which code for non-structural movement (NSm) and precursor of glycoproteins (GP) (Kormelink et al., 1992). GP is post-translationally cleaved into Gn and Gc glycoproteins (Adkins et al., 1996). S RNA also contains two ORFs separated by an IGR, which code for non-structural suppressor of silencing (NSs) and nucleocapsid (N) proteins (de Haan et al., 1990). The virion core contains ribonucleoproteins (RNPs) consisting of RNA segments tightly associated with N protein (Ie, 1971; Kormelink, 2011; Li et al., 2014). Encapsidated tospovirus RNA genome segments with several copies of RdRP are collectively bound by a lipid envelope, which is decorated by Gn and Gc glycoprotein projections (van Poelwuk et al., 1993; Kikkert et al., 1999).

Tomato spotted wilt virus (TSWV) is the best-studied tospovirus. Molecular biology of TSWV infection has been studied using various model plant and animal systems (Kikkert et al., 2001; Lewandowski and Adkins, 2005; Snippe et al., 2005, 2007a,b; Paape et al., 2006; Ribeiro et al., 2008, 2009). TSWV replication, transcription and particle assembly takes place in the cytoplasm (Ie, 1971; Goldbach and Peters, 1996; Kikkert et al., 1999). In host plants, viral RNPs in association with NSm are transported from initially infected cells to neighboring cells through plasmodesmata (PD), assisting progression of the infection (Storms et al., 1995; Soellick et al., 2000). Recently, molecular interactions of viral and host proteins have been reported for TSWV and several other tospoviruses (Paape et al., 2006; Feng et al., 2013, 2016; Singh and Savithri, 2015). These findings point to significant complexity in the tospovirus infection process and unexplored unique characteristics for some tospovirus proteins beyond TSWV.

Although CaCV has been identified more than a decade ago, it has not been widely studied. Hence, to understand the molecular basis of CaCV infection process while constrained by the lack of a reverse genetics system for tospoviruses, we determined intracellular localization and interactions of its proteins and discuss these data in the context of viral replication, particle assembly, and intercellular movement. Furthermore, we have experimentally demonstrated the functions of CaCV non-structural proteins NSm and NSs that are involved in cell-to-cell movement and RNA silencing suppression, respectively.

## Materials and Methods

### Plasmid Constructs

All CaCV ORFs except that coding for RdRP were amplified by reverse transcription polymerase chain reaction (RT-PCR) and cloned into plant expression destination vectors using Gateway^TM^ technology. Briefly, cDNA was synthesized from total RNA extracted from CaCV (QLD-3432)-infected capsicum using SuperScript III First-Strand Synthesis SuperMix (Life Technologies) and random primers. CaCV ORFs encoding N, NSs, NSm, Gc, and Gn proteins were amplified with gene-specific primers using Phusion High Fidelity polymerase (Finnzymes). To facilitate subsequent Gateway cloning, primer sequences were flanked with *attB* recombination sites at 5′ ends. PCR amplicons were gel-purified (Wizard gel purification kit, Promega), cloned into pDONR221 using BP Clonase II Enzyme Mix (Life Technologies) and transformed into chemically competent Omnimax *Escherichia coli* (Life Technologies) using heat shock method. Recombinant colonies were selected on Luria-Bertani (LB) agar containing 50 mg/L kanamycin. Plasmid DNA was extracted from recombinant bacterial cultures using GeneJet plasmid purification kit (Thermo Fisher Scientific) and sequenced. Sequence-validated entry clones were recombined into C-series of pSITE and pSITE II destination vectors (Chakrabarty et al., 2007; Martin et al., 2009) as fusions to green fluorescent protein (GFP), red fluorescent protein (RFP) and/or Flag peptide for localization studies and to yellow fluorescent protein (YFP) fragments for bimolecular fluorescence complementation (BiFC) assays using LR Clonase II Enzyme Mix (Life Technologies). Recombinant pSITE vectors carrying CaCV ORFs were confirmed by colony PCR (AmpliTaq Gold-Fast PCR, Applied Biosystems, Thermo Fisher Scientific) and were individually transformed into *Agrobacterium tumefaciens* LBA 4404.

### Agroinfiltration and Live Cell Imaging

CaCV fusion proteins were transiently expressed in plants using leaf agroinfiltration to determine intracellular localization and interactions. Agrobacterium suspensions at 0.7–0.8 optical density (OD_600_) were infiltrated into *Nicotiana benthamiana* epidermal leaf cells as previously described (Tsai et al., 2005). For localization studies, *N. benthamiana* wild-type or RFP fused to histone 2B (RFP-H2B) transgenic marker plants, or capsicum cv. Yolo Wonder were used, whereas for BiFC protein–protein interaction assays, cyan fluorescent protein fused to histone 2B (CFP-H2B) transgenic marker plants were used (Martin et al., 2009). To visualize intracellular structures, mCherry-ER (endoplasmic reticulum) and mCherry-Golgi marker plasmids (Nelson et al., 2007) transformed into *A. tumefaciens* LBA 4404 were co-infiltrated with cultures carrying CaCV fusion protein constructs. Callose deposits at PD were stained with 0.033 mg/mL aniline blue fluorochrome (Biosupplies, Australia) by infiltration into leaves 30 min before visualization. For BiFC assays all viral proteins were expressed as C-terminal fusions to the amino- or carboxy-terminal portions of YFP using pSITE-BiFC-nEYFP and pSITE-BiFC-cEYFP vectors (Martin et al., 2009). Leaf sections were viewed using Zeiss LSM-700 (Carl Zeiss) confocal laser scanning microscope (CLSM) at 2 days post infiltration (dpi). Images were acquired as CZI files and processed using Zen Lite 2012 and ImageJ software (Schneider et al., 2012).

### Protoplast Isolation and Imaging of NSm Expression

Protoplasts were isolated from *N. benthamiana* leaves according to a protocol kindly provided by Dr. Richard Kormelink (Wageningen University, The Netherlands) with slight modifications. Briefly, leaves from greenhouse-grown, 4-weeks old *N. benthamiana* were co-agroinfiltrated with pSITE constructs carrying RFP-NSm and free eGFP (Chakrabarty et al., 2007). Infiltrated plants were incubated under constant light for 22 h at room temperature and protoplasts were isolated from 2 g of infiltrated tissue. Harvested leaves were surface-sterilized by immersing in 70% ethanol for 10 s, followed by three washes in sterile MilliQ water. After removal of mid rib, leaves were sliced into 1–2 mm strips and macerated overnight in the dark at 4°C in 50 mL of an enzyme solution containing 0.6 g cellulase (Onozuka R-10), 30 mg Macerozyme R-10 (*Phyto*Technology Laboratories), and 4.55 g mannitol. Released protoplasts were filtered through a nylon cell strainer with 70 μm diameter pores (Falcon, Corning brand) and collected on a 20% sucrose cushion after centrifugation for 10 min at 100 ×*g*. Protoplasts were collected from the interface and resuspended in 0.6 M mannitol. The suspension was centrifuged again to collect protoplasts and resuspended in mannitol solution to adjust volume and concentration. Protoplast viability was tested by staining with 1% Evan’s Blue (Sigma–Aldrich). Viable protoplasts were counted using a haemocytometer (Weber, England) using a light microscope with 10 × 10 magnification (Olympus BH2). Protoplast suspension was spotted on poly-L-lysine coated glass slides (Sigma–Aldrich) and incubated with constant light at room temperature. Protoplasts were observed with Zeiss LSM-700 (Carl Zeiss) CLSM starting from 4 to 8 h of incubation. Images were acquired using 40x lens as CZI files and processed using Zen Lite 2012 and ImageJ software (Schneider et al., 2012).

### Immunofluorescent Detection of NSs Protein in Protoplasts

Nuclear localization of NSs was validated by immunofluorescence microscopy. Protoplasts were isolated from RFP-H2B transgenic *N. benthamiana* leaf tissue that transiently expressed Flag-tagged CaCV NSs 22 h after agroinfiltration. As control, protoplasts were isolated from plants agroinfiltrated with pSITE-Flag vector. Protoplast suspension was spotted on poly-L-lysine coated glass slides. NSs was detected using a mouse monoclonal antibody (MAb) (1:1000) directed against NSs of watermelon silver mottle virus (WSMoV) serogroup viruses (Chen et al., 2006) following a previously described protocol with slight modifications (Hibi et al., 1975; Kikkert et al., 1997). A mouse IgG1 antibody (1:1000) was used as non-specific antibody control. Antigen-antibody complexes were detected by goat anti-mouse IgG (H + L) conjugated with Alexa Fluor 488 (1:300, Life Technologies). Slides were examined with a Zeiss LSM-700 (Carl Zeiss) CLSM.

### Virus Movement *Trans*-complementation

Cell-to-cell movement function of NSm was demonstrated using a turnip vein clearing virus (TVCV)-based movement *trans*-complementation system (Mann et al., 2016) derived from a pro-vector system consisting of three modules; 5′ module pICH17388: TVCV RdRP and TVCV P30 MP; 3′ module pICH7410: GFP reporter and tobacco mosaic virus (TMV) non-translated region and pICH14011: integrase (Marillonnet et al., 2004, 2005; Giritch et al., 2006). This system was modified to be movement defective by introducing a mutation in P30 MP (Mann et al., 2016). NSm or NSm mutant (ΔNSm) constructs fused to RFP were co-expressed with movement-defective TVCV system by agroinfiltration into *N. benthamiana* epidermal leaf cells following the method described by Mann et al. (2016). Functional and mutated TVCV P30 (Mann et al., 2016) and TSWV NSm (QLD-1255 isolate) fused to RFP were used as positive controls. Empty RFP vector was used as negative control. Images were acquired 7 days after agroinfiltration using a Zeiss LSM-700 (Carl Zeiss) CLSM. At least 50 GFP foci per construct per experiment were observed and cells expressing GFP were counted. The efficiency of cell-to-cell movement was estimated by counting number of adjacent cells expressing GFP.

### Generation of NSm Mutants

Sequence-validated CaCV and TSWV NSm entry clones were used as templates to generate movement-defective NSm (ΔNSm) constructs using Q5^®^Site-Directed Mutagenesis kit (New England Biolabs) following manufacturer’s instructions. Primers for mutagenesis were designed using NEBaseChanger (New England Biolabs) online following the recommendations for substitution primers. Mutations in entry clones were confirmed by sequencing. Mutants were recombined into pSITE-RFP-C1 vector and transformed into *A. tumefaciens* LBA 4404.

### Suppression of *gfp* Silencing

Entry clones of CaCV NSs in pDONR221 were recombined into pSITE-Flag-C1 destination vector (Chakrabarty et al., 2007). Similarly, a recombinant pSITE vector carrying Flag-TSWV NSs was constructed from TSWV QLD-1255 isolate as positive control. Recombinant vectors were transformed into *A. tumefaciens* LBA4404. Agrobacteria harboring recombinant pBIN vector carrying mGFP5-ER gene were used as inducer of RNA silencing. Agrobacteria carrying Flag-NSs and mGFP5-ER constructs were co-infiltrated with 1:1 ratio into GFP-expressing *N. benthamiana* line 16c (Ruiz et al., 1998). Local and systemic silencing assays were independently performed at least three times. GFP fluorescence was monitored at 3 and 7 dpi for local silencing and 14 and 21 dpi for systemic silencing using a blue light (HL32T Dark Reader hand lamp, Clare Chemicals Research). Images were acquired using Canon EOS 600D digital camera equipped with a blue light filter. Formation of red zone surrounding infiltrated patches was observed using a long wavelength Black Ray model B 100AP UV lamp (UVP, Upland, CA, USA) at 365 nm. Images were acquired using Canon EOS 450D camera equipped with a GFP filter.

### Protein Expression Analysis

Transient expression of two selected viral proteins fused to GFP, N, and NSs following agroinfiltration was validated by western blot. Soluble proteins from agroinfiltrated leaf tissues at 2 dpi (for localization assay) or 3 dpi (suppression of RNA silencing assay) were extracted in protein sample buffer (Laemmli, 1970) and separated on a SDS-12% polyacrylamide gel. Proteins were transferred to PVDF membrane (Millipore) and detected using mouse anti-GFP antibodies (1:1000, Roche Life Science) or mouse monoclonal anti-Flag M2 clone 2 antibody (1:2000, Sigma–Aldrich) followed by rabbit anti-mouse IgG horseradish peroxidase conjugate (1:25,000), Pierce ECL Plus chemiluminescent substrate, and exposure to x-ray film (Fujifilm). Protein sizes were estimated using pre-stained Page Ruler protein ladder (Thermo Fisher Scientific).

### *In silico* Sequence Analyses

Deduced amino acid sequences of CaCV proteins were analyzed for potential nuclear localization (import) and nuclear export signals using cNLS Mapper (Kosugi et al., 2009) and NetNES 1.1 server (La Cour et al., 2004). Secondary structure prediction and alignment of CaCV (KM589494), TSWV NSm (QLD-1255, unpublished), TMV P30 (NC_001367.1), and TVCV P30 (U03387) were carried out using PROMALS3D online (Pei and Grishin, 2014). NSm sequences were analyzed for predicted coiled-coil domains using an online server (McDonnell et al., 2006).

## Results

### Intracellular Localization of CaCV Proteins *In planta*

**FIGURE 1:**
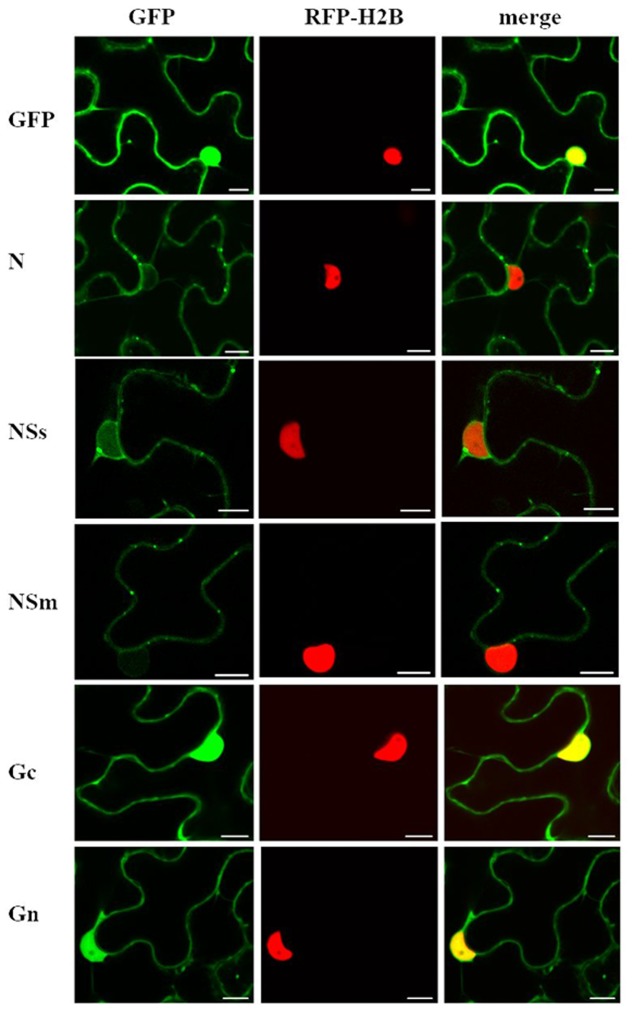
**Intracellular localization of transiently expressed free green fluorescent protein (GFP) or capsicum chlorosis virus (CaCV) proteins fused to GFP.** CaCV proteins N, NSs, NSm, Gc, Gn were individually expressed from pSITE vectors that were agroinfiltrated into transgenic red fluorescent protein (RFP)-histone 2B (H2B) *Nicotiana benthamiana* leaf epidermal cells. Images were acquired after 2 days using a confocal microscope at 10 × 25 magnification. Left column, GFP channel; center column, RFP channel; right column, merged images. Bars, 10 μm.

To determine intracellular localization of viral proteins, we transiently expressed all individual CaCV proteins except RdRP as fusions to the C-terminus of GFP and visualized fusion protein accumulation using CLSM. Expression of GFP-N and GFP-NSs fusion proteins was confirmed by western blot at 3 dpi (data not shown). Experimental evidence for expression of all GFP fusion proteins is provided by confocal microscopy showing altered intracellular localization compared to free GFP (**Figure 1**).

In *N. benthamiana*, all ectopically expressed CaCV proteins localized to the cell periphery near ER membranes (**Figure 1** and Supplementary Figure 1). Free GFP could be seen in both the cell periphery and in the nucleus. GFP:N formed varying sized aggregates in the cell periphery and perinuclear region. GFP:NSs showed slightly punctate distribution in the cell periphery. In addition, NSs also accumulated in the nucleus in agreement with a predicted moderately strong bipartite NLS (cNLS mapper score 4.1). However, TSWV NSs that is thought to be cytoplasmic yielded a similar cNLS score of 4.4. GFP:NSs was observed in 86% of nuclei in three independent experiments with a total of 50 nuclei observed in each experiment. Since accumulation of NSs in the nucleus was unexpected considering previous localization studies of NSs of other tospoviruses, we captured three-dimensional Z-stack images at various depths to validate NSs nuclear localization (Supplementary Figure 2). Composite images confirmed that NSs indeed accumulated inside the nucleus, but not in the nucleolus that appeared as a dark spot (Supplementary Figure 2-V). GFP:NSm showed discontinuous punctate spots in the cell periphery. GFP:Gc and GFP:Gn showed similar localization profiles with smooth distribution in the cell periphery and also accumulation in the nucleus as supported by predicted canonical NLSs. The cNLS mapper software predicted two monopartite NLSs (scores 5.5 and 4.0) and two bipartite NLSs (scores 7.6 and 4.1) for Gc and two bipartite NLSs (score 4.1) for Gn. Twenty nuclei each observed in three independent experiments showed 100% GFP:Gc and GFP:Gn nuclear localization.

Localization of CaCV proteins was validated in wild-type *N. benthamiana* and in capsicum plants (Supplementary Figures 1A,B), both susceptible host plant species for CaCV. ER membranes were visualized as an internal marker by co-expression of ER-targeted mCherry RFP. All CaCV proteins showed a similar localization pattern as that in transgenic RFP-H2B *N. benthamiana* cells (compare **Figure 1** and Supplementary Figure 1). GFP:NSs was consistently observed in >80% of nuclei in cells of wild-type and transgenic *N. benthamiana* and wild-type capsicum.

### CaCV NSs Accumulates in the Nucleus

**FIGURE 2:**
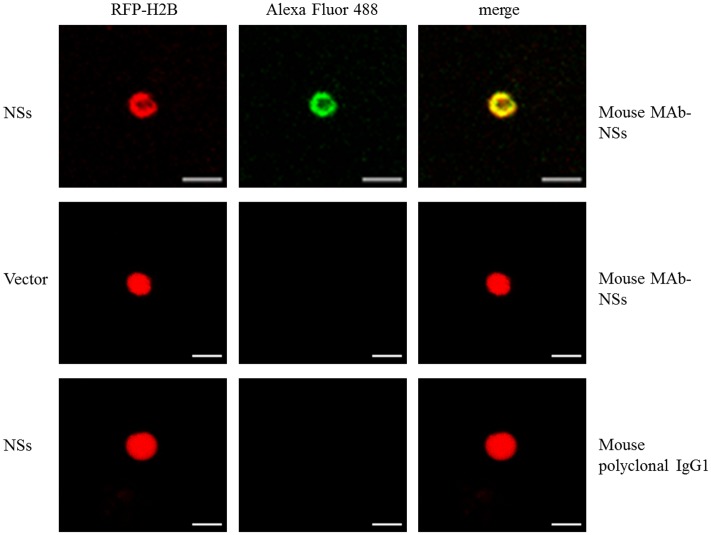
**Immunofluorescent detection of transiently expressed CaCV NSs protein in nuclei of RFP-H2B *N. benthamiana* protoplasts.** Transgenic RFP-H2B nuclear marker protoplast preparations expressing NSs or empty vector were incubated with mouse monoclonal antibody (MAb), raised against NSs or non-specific mouse IgG1 antibody and detected using goat anti-mouse IgG conjugated with Alexa Fluor 488. Fluorescence of Alexa Fluor 488 was viewed using the GFP channel and RFP using the red channel. Bar, 10 μm.

We validated nuclear accumulation of CaCV NSs by immunofluorescence using a MAb directed against a common epitope in the NSs of tospovirus members of the WSMoV serogroup (Chen et al., 2006) in protoplasts of RFP-H2B nuclear marker *N. benthamiana* that transiently expressed CaCV NSs. Controls used in this experiment to validate specific MAb binding to NSs included agroinfiltrated empty vector and a mouse IgG1 polyclonal antibody for detection. NSs localization was determined by indirect immunofluorescence. Bright green fluorescence of Alexa Fluor 488 conjugate overlapped with red (RFP) nuclei of protoplasts expressing NSs (**Figure 2**). In contrast, we did not detect any fluorescence in the nuclei or the cytoplasm of control protoplasts or when mouse IgG was used for detection. Nuclear localization of NSs was seen in >95% of a total of 50 nuclei observed in two independent experiments based on the number of green fluorescing nuclei.

### Golgi Complex Association of CaCV Glycoproteins

**FIGURE 3:**
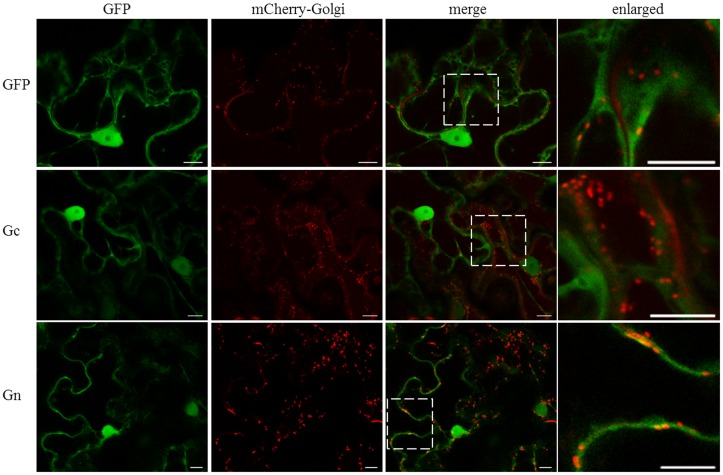
**Intracellular localization of free GFP and CaCV Gc and Gn glycoproteins fused to GFP, relative to mCherry-Golgi marker.** GFP, viral fusion proteins, and mCherry-Golgi marker were transiently expressed following co-agroinfiltration of gene expression constructs into *N. benthamiana* leaf epidermal cells. Enlarged sections of images are highlighted with dotted boxes. Images were acquired 2 days after agroinfiltration using a confocal microscope at 10 × 25 magnification. Bar, 10 μm.

We tested the potential association of individual CaCV glycoproteins with the Golgi complex by transient co-expression of GFP:Gc and/or GFP:Gn with mCherry-Golgi marker (Nelson et al., 2007). With this marker Golgi stacks could be seen as round shaped disks in the cytoplasm and along the cell periphery (**Figure 3**). In addition mCherry-Golgi weakly labeled the ER. Both GFP:Gc and GFP:Gn appeared largely non-Golgi associated, however, some orange colored spots appeared with GFP:Gn due to co-localization of GFP and mCherry fusion proteins in merged images (**Figure 3**). Light orange colored spots were also visible with free GFP control and GFP:Gc co-localized with mCherry-Golgi. When enlarged, co-localization spots of Gn could be more clearly distinguished with distinct orange color compared to free GFP and Gc spots. Therefore, it appears that Gn, but not Gc localizes infrequently within Golgi stacks. To determine whether co-expression of both glycoproteins affects their localization, we transiently co-expressed the glycoproteins as GFP and/or Flag fusions. Co-expression of Gc and Gn did not show an altered localization profile compared to the individual fusion proteins (data not shown).

### CaCV NSm Protein Localizes at Plasmodesmata

**FIGURE 4:**
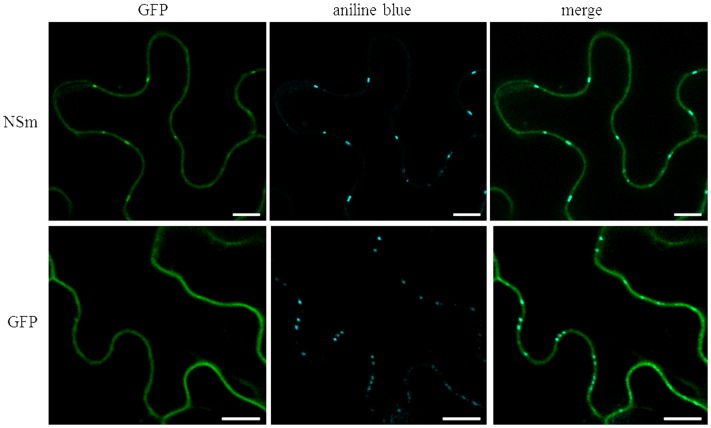
**Co-localization of CaCV NSm protein with plasmodesmata (PD) marker dye, aniline blue.** *N. benthamiana* leaves transiently expressing GFP:NSm or free GFP were infiltrated with aniline blue fluorochrome solution. Images were taken 2 days after agroinfiltration using a confocal microscope at 10 × 25 magnification. Aniline blue was false color imaged as cyan to increase contrast. Bars, 10 μm.

Based on well-studied NSm proteins of other tospoviruses, we predicted that CaCV NSm is involved in viral cell-to-cell movement. This hypothesis is supported by NSm protein localization at PD as evidenced by discontinues punctate spots in the cell periphery (**Figure 1** and Supplementary Figure 1). We validated PD localization in *N. benthamiana* cells transiently expressing CaCV GFP:NSm by confocal microscopy at 2 dpi relative to the location of aniline blue fluorochrome which stains callose-rich regions at PD. GFP:NSm punctate spots on the cell periphery showed perfect co-localization with aniline blue dye indicating PD localization of CaCV NSm (**Figure 4**).

### Intracellular Interactions of CaCV Proteins *In planta*

**FIGURE 5:**
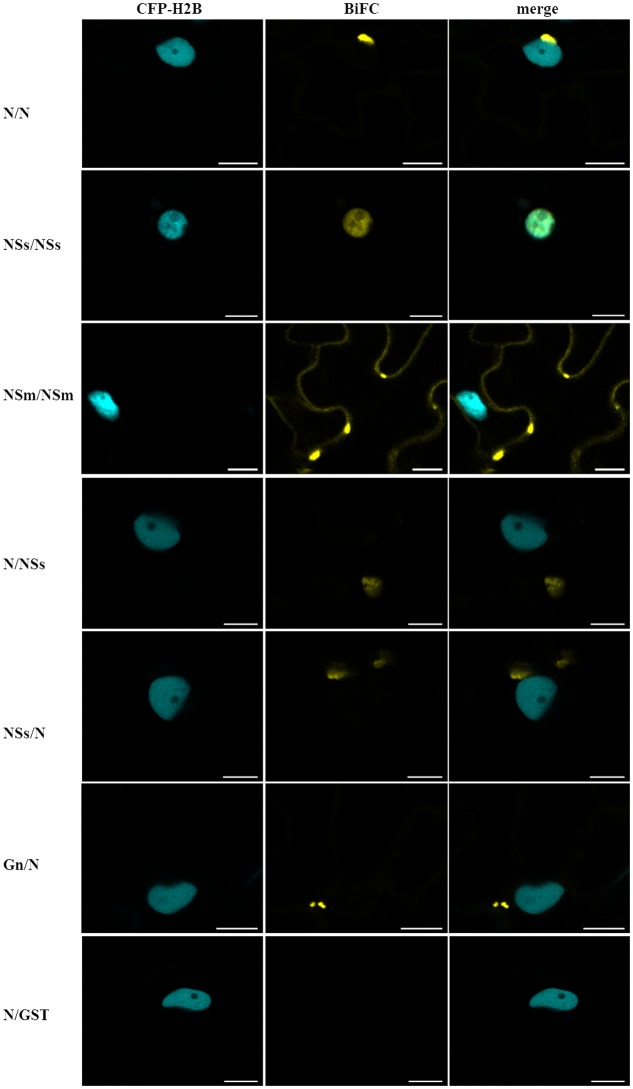
**Bimolecular fluorescence complementation (BiFC) to identify CaCV protein–protein interactions.** Viral proteins were transiently expressed as fusions to yellow fluorescent protein (YFP) N- or C-terminal fragments in pSITE-BiFC vectors following agroinfiltration into transgenic *N. benthamiana* expressing cyan fluorescent protein fused to histone 2B (CFP-H2B). Images of leaf epidermal cells were acquired after 2 days using a confocal microscope at 10 × 25 magnification. YFP fragments in interacting protein combinations are indicated in the order n-YFP/c-YFP. Glutathione-*S*-transferase (GST) was used as non-binding control in all combinations, but only one example is shown. Bars, 10 μm.

We used BiFC to determine CaCV protein–protein interactions and localization in all pair-wise combinations. Individual proteins were transiently expressed in transgenic CFP-H2B plants as fusions to the C-terminus of YFP n- and c-terminal fragments. Glutathione-*S*-transferase (GST) was used as non-binding control. BiFC interactions were seen as restoration of YFP fluorescence (**Figure 5**). We observed homotypic (self) interactions of N, NSs, and NSm. N–N interactions appeared as abundant aggregates in diverse sizes in the cytoplasm and perinuclear region, similar to N protein localization (see **Figure 1** and Supplementary Figure 1). NSs self-interaction was observed exclusively inside the nucleus. NSm homotypic interactions were seen along the cell periphery as discontinuous punctate spots. We also detected heterotypic interactions involving N protein in the cytoplasm. N interacted with NSs in both orientations and with Gn only in the Gn-N orientation (**Figure 5**). No other interactions were observed by BiFC in either orientation. None of the CaCV proteins showed any interaction with GST in either orientation (**Figure 5** and data not shown).

### CaCV and TSWV NSm *Trans*-complement Cell-to-Cell Movement of a Movement-defective Tobamovirus Replicon

We provide experimental evidence that both CaCV and TSWV NSm function as cell-to-cell movement proteins in a tobamovirus replicon *trans*-complementation assay. NSm proteins fused to RFP (so their expression could be verified by CLSM) were transiently expressed in *N. benthamiana* epidermal leaf cells together with a movement-defective TVCV replicon (TVCVΔMP) composed of three modules; defective movement module, GFP reporter module, and integrase module. Complementation of TVCVΔMP movement was observed by confocal microscopy as spread of the GFP reporter replicon across five or more adjacent cells. Empty RFP vector was used as negative control and homologous P30:RFP as positive control.

**FIGURE 6:**
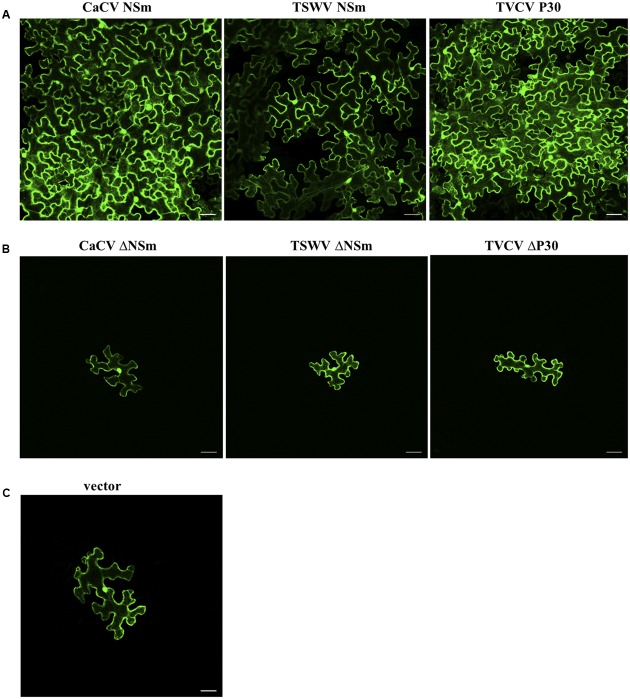
***Trans*-complementation by CaCV NSm of cell-to-cell movement of GFP expressing turnip vein clearing virus (TVCV) replicon.** Functional **(A)** or dysfunctional (Δ, **B**) movement proteins were transiently expressed together with movement-defective TVCV replicon system in *N. benthamiana* leaf epidermal cells. Images were taken 7 days after agroinfiltration using confocal microscope at 10 × 10 magnification. Tomato spotted wilt virus (TSWV) NSm, TVCV P30 and empty vector **(C)** were used as controls. Bars, 10 μm.

Co-expression of TVCVΔMP modules with CaCV or TSWV NSm showed many GFP-expressing cell clusters comprising five cells or more, similar to the TVCV P30 control at 7 dpi (**Figure 6A**). By contrast, when TVCVΔMP was co-expressed with empty RFP vector, GFP expression was limited to single cells (**Figure 6C**). These data provide evidence that tospovirus NSm was able to facilitate cell-to-cell movement of a movement-defective TVCV replicon. However, cell-to-cell movement pattern of TVCVΔMP replicon appeared different when complemented by CaCV NSm versus TSWV NSm. CaCV NSm *trans*-complemented TVCVΔMP movement radially in all directions generating large clusters of cells similar to TVCV P30. On the other hand, TSWV NSm *trans*-complementation often led to movement in a single direction generating a line of GFP-expressing cells and rarely led to radial clusters (data not shown). Efficiencies of movement *trans*-complementation by TVCV P30, CaCV NSm, and TSWV NSm were 65, 51, and 36%, respectively (**Table 1**).

**Table 1:** Cell-to-cell movement *trans*-complementation of turnip vein clearing virus (TVCV) movement-defective replicon (TVCVΔMP) by functional and dysfunctional (Δ) tospovirus NSm proteins.

TVCVΔMP+	1–2 cells	3–4 cells	≥5 cells	Total foci counted^∗^	Percent (%) efficiency of movement *trans*-complementation (≥5 cells)
RFP-CaCV NSm	48	50	104	202	51
RFP-CaCV ΔNSm	160	3	0	163	0
RFP-TSWV NSm	52	46	55	153	36
RFP-TSWV ΔNSm	145	8	0	153	0
TVCV P30- RFP	40	29	129	198	65
TVCV ΔP30- RFP	144	7	0	151	0
Empty RFP- vector	146	8	0	154	0

*^∗^Total green fluorescent protein (GFP) foci counted in three independent experiments.*

Secondary structure prediction showed tospovirus MPs contain the conserved 30K protein-specific LxD/N_50-70_G motif. To confirm specificity of NSm movement function, we generated mutant RFP-NSm (RFP-ΔNSm) constructs by replacing D (aspartic acid) residue with A (alanine) in the conserved motif. At 7 dpi, TVCVΔMP co-expressed with CaCV or TSWV RFP-ΔNSm or ΔP30-RFP showed limited GFP expression confined to majority of 1–2 isolated cells similar to vector control (**Figure 6B** and **Table 1**). Taken together these data provide strong evidence that CaCV and TSWV NSm possess cell-to-cell movement activity that was able to *trans*-complement movement of a dysfunctional tobamovirus replicon, and mutation of the essential D residue in the LxD/N_50-70_G motif specifically eliminated this movement activity.

**FIGURE 7:**
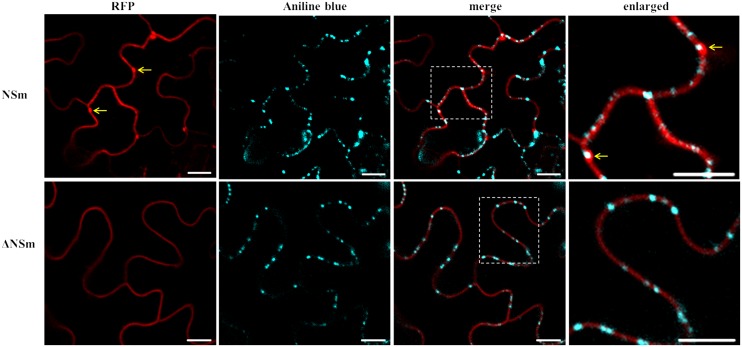
**Intracellular localization of CaCV NSm protein and PD.** RFP fusions of functional (NSm) and dysfunctional (ΔNSm) protein-expressing *N. benthamiana* leaves were stained with aniline blue fluorochrome to visualize PD. Enlarged sections of images are highlighted with dotted boxes. Co-localization of NSm aggregates and aniline blue are indicated with arrows. To increase contrast, aniline blue fluorescent images were false colored as cyan. Images were taken at 2 days post infiltration (dpi) using a confocal microscope at 10 × 25 magnification. Bars, 10 μm.

To determine whether substitution of D residue in LxD/N_50-70_G motif also effects PD localization, *N. benthamiana* leaves transiently expressing CaCV RFP-NSm or -ΔNSm were infiltrated with aniline blue to stain callose at PD. Wild-type RFP-NSm localized to the cell periphery in punctate spots (**Figure 7**) similar to GFP-NSm (**Figure 1**), whereas RFP-ΔNSm was instead smoothly distributed along the cell periphery (**Figure 7**). RFP-NSm aggregates perfectly overlapped with aniline blue spots indicating PD localization. Collectively, these data confirm that mutation of D_155_ residue in CaCV LxD/N_50-70_G motif eliminates PD localization and cell-to-cell movement activity of NSm protein, suggesting that CaCV NSm is a 30K-like viral movement protein.

### CaCV NSm Forms Tubular Structures Protruding from Protoplasts

**FIGURE 8:**
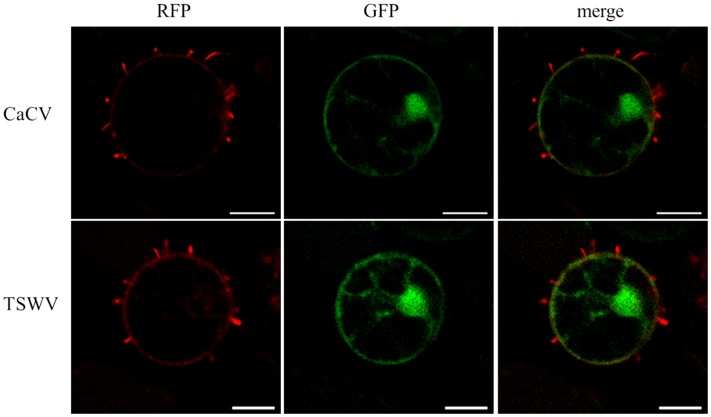
**Tubule-like structures protruding from protoplasts expressing tospovirus NSm proteins.** Protoplasts were prepared 22 h after agroinfiltration from *N. benthamiana* leaves transiently expressing CaCV or TSWV NSm fused to RFP plus free GFP. Images were taken using a confocal microscope at 10 × 40 magnification after 5 h incubation of freshly prepared protoplasts at room temperature with constant light. Bars, 10 μm.

We observed tubule-like projections on the surface of protoplasts that had been isolated from *N. benthamiana* leaves that transiently expressed RFP-NSm (CaCV or TSWV). TSWV NSm was used as a positive control and free GFP expression was used to highlight location of cellular structures, ER, and nucleus. Tubular structures appeared after 5 h incubation of protoplast suspensions on poly-L-lysine coated slides (**Figure 8**). Often, tubular structures looked like short projections, but occasionally longer tubules were seen. These results provide evidence that CaCV NSm, like its TSWV counterpart is capable of forming tubular structures, which may facilitate cell-to-cell movement of the virus in infected plant cells.

### CaCV NSs Suppresses *gfp* Silencing Locally and Systemically

**FIGURE 9:**
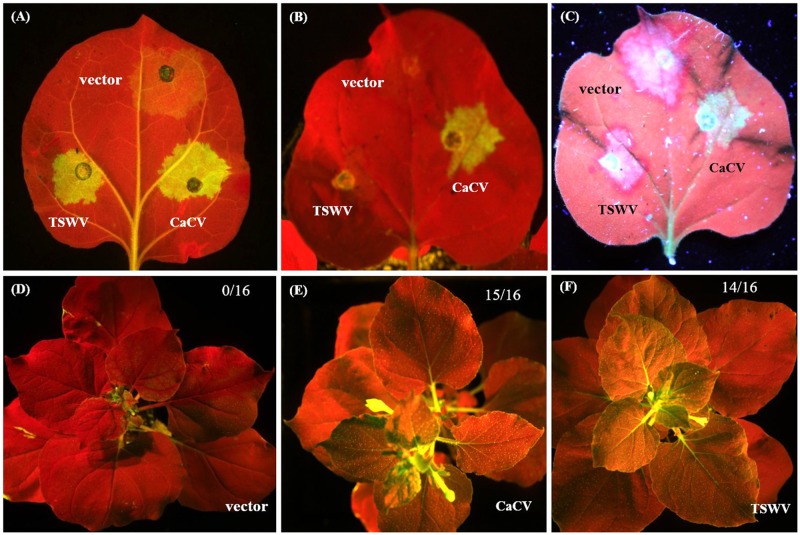
**Suppression of local and systemic *gfp* silencing by CaCV NSs protein.** For local silencing assay, leaf patches of GFP-expressing *N. benthamiana* line 16c were agroinfiltrated with mGFP5-ER plus either CaCV NSs, TSWV NSs or empty vector and images were taken at day 3 **(A)** and day 7 **(B)**. Formation of red halo surrounding infiltrated patches was viewed at 7 dpi **(C)**. For systemic silencing assay, line 16c plants were agroinfiltrated with mGFP5-ER plus either empty vector **(D)**, CaCV NSs **(E)**, or TSWV NSs **(F)** and images were taken at day 21. Number of GFP-expressing plants (silencing suppressed) per total number of plants tested are shown in the top right of each image. A Canon EOS camera containing a blue filter was used to take images **(A,B,D–F)** of plants exposed to blue light and image **(C)** was captured by exposing the same leaf shown in image **(B)** to long wavelength UV light using a Canon EOS camera equipped with a GFP filter.

We demonstrate that CaCV NSs functions as a suppressor of RNA silencing using well-established GFP reporter assays (Johansen and Carrington, 2001). CaCV NSs was co-expressed with GFP silencing inducer, mGFP5-ER in transgenic *N. benthamiana* line 16c, which constitutively expresses mGFP5-ER. The known suppressor TSWV NSs and pSITE-Flag vector were used as positive and negative controls, respectively. Transient expression of Flag-tagged NSs proteins was confirmed by western blot at 3 dpi (data not shown). Co-expression of CaCV NSs with GFP reporter gene enhanced visible GFP expression at 3–4 days after infiltration locally in the infiltrated leaf patch (**Figure 9A**). GFP fluorescence was strong and comparable to that seen with TSWV NSs. Leaf patches infiltrated with empty vector showed significantly diminished GFP fluorescence, indicating silencing of the reporter gene. This data confirms that CaCV NSs is a local RNA silencing suppressor. GFP fluorescence in the CaCV NSs expressing patch remained bright at 7 dpi, while *gfp* reporter in TSWV NSs and vector leaf patches were silenced (**Figure 9B**). To investigate if NSs has an effect on the short-distance spread of *gfp* silencing, the formation of an indicative ‘red zone’ was monitored at 7 dpi. Wide red zones were clearly visible surrounding the leaf patches expressing TSWV NSs and vector (**Figure 9C**). At that time point, local GFP expression in the infiltrated leaf patch had ceased/was silenced (TSWV NSs silencing suppression was overcome), while systemic movement of the silencing signal had been initiated. By contrast, a narrow red zone was visible surrounding the leaf patch expressing CaCV NSs. This data indicates that CaCV NSs delays or interferes with the short-distance spread of the RNA silencing signal.

To investigate systemic silencing activity of CaCV NSs, GFP fluorescence in upper non-infiltrated leaves was monitored in *N. benthamiana* line 16c plants that had been agroinfiltrated with NSs constructs or empty vector plus mGFP5-ER. Leaves were considered systemically silenced if complete or partial chlorophyll auto fluorescence (red) was observed whereas leaves were considered non-silenced/ suppressed if GFP fluorescence resembled that of the mock (buffer-infiltrated) control plants. At 21 dpi, in two independent experiments all plants infiltrated with empty vector and mGFP5-ER constructs (16/16) showed GFP silencing in upper non-infiltrated leaves (**Figure 9D**), whereas in most plants that were agroinfiltrated with constructs expressing mGFP5-ER plus CaCV NSs (15/16) or TSWV NSs (14/16) systemic silencing was suppressed and GFP expression was observed (**Figures 9E,F**). This data indicates that CaCV NSs suppresses GFP silencing systemically with a similar efficiency as TSWV NSs.

## Discussion

Tomato spotted wilt virus is the best-studied tospovirus in terms of particle morphology, genome organization, replication and transcription strategies and therefore TSWV represents the prototype tospovirus (Kormelink et al., 2011). However, recent studies of TSWV and other tospoviruses have provided evidence for novel or previously unexplored tospovirus features. For instance, TSWV N protein has been shown to traffic on the actin/ER network, a novel property of a plant virus capsid protein (Feng et al., 2013). Studies on groundnut bud necrosis virus (GBNV) unraveled a novel feature of tospovirus NSm, which remodels ER networks to form vesicles to facilitate viral movement (Singh and Savithri, 2015). CaCV is an emerging tospovirus which is serologically and phylogenetically distinct (belongs to a different clade) from TSWV (McMichael et al., 2002; Oliver and Whitfield, 2016). Molecular characteristics of CaCV are largely unknown. In this study, we investigated properties of CaCV structural and non-structural proteins in comparison to those of TSWV to gain a better understanding of their involvement in the tospovirus infection cycle. We determined intracellular localizations and interactions of CaCV proteins in living plant cells and characterized the functions of the two non-structural proteins.

**FIGURE 10:**
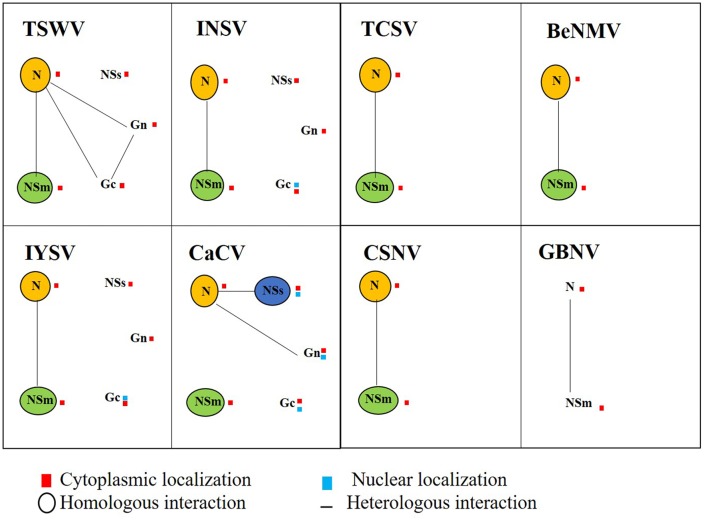
**Schematic map diagram of integrated localization and interaction data for tospovirus proteins determined in this study and compiled from the scientific literature for TSWV, impatiens necrotic spot virus (INSV), tomato chlorotic spot virus (TCSV), bean necrotic mosaic virus (BeNMV), iris yellow spot virus (IYSV), CaCV, chrysanthemum stem necrosis virus (CSNV), and groundnut bud necrosis virus (GBNV).** Tospovirus proteins N, NSs, NSm, Gc, and Gn are indicated with homotypic interactions shown by circles and heterotypic interactions by connecting lines. Location of the interaction is indicated by red (cytoplasm) and/or blue (nucleus) squares next to the protein symbol.

Tospoviruses studied so far are known to replicate in the cytoplasm of infected host plant cells (Poelwijk et al., 1995; Goldbach and Peters, 1996). Our data regarding CaCV protein intracellular localization and interactions provide evidence that CaCV also replicates in the cytoplasm. CaCV structural (N, Gc, Gn) and non-structural (NSs, NSm) proteins accumulated in the cytoplasm when transiently overexpressed. Cytoplasmic localization of viral proteins is common among tospoviruses (Lacorte et al., 2007; Kormelink et al., 2011; Dietzgen et al., 2012; Leastro et al., 2015; Tripathi et al., 2015a) and CaCV is no exception. We observed comparable intracellular localization patterns of CaCV proteins in the susceptible host plants *N. benthamiana* and capsicum, suggesting that protein localization may be independent of the host plant species. N protein localization showed aggregates abundantly dispersed in the cell periphery and in the perinuclear region in association with ER. As a structural protein that binds to and protects viral genomic RNA (Ie, 1971; Li et al., 2014), it is not surprising to see N protein aggregates in the cytoplasm of agroinfiltrated cells. BiFC showed that these aggregates were the result of N protein self-interaction. Self-interaction of N protein has previously been reported for several tospoviruses (**Figure 10**) including TSWV, CaCV-AIT (Thailand), and INSV (Lacorte et al., 2007; Zilian and Maiss, 2011; Dietzgen et al., 2012) and more recently for, bean necrotic mosaic virus (BeNMV), chrysanthemum stem necrosis virus (CSNV), iris yellow spot virus (IYSV), and tomato chlorotic spot virus (TCSV) (Leastro et al., 2015; Tripathi et al., 2015a). It is likely that CaCV N protein aggregates in association with ER are N protein inclusions that traffic along the ER and actin network as was shown for TSWV (Feng et al., 2013).

It has been shown previously that the final intracellular destination of TSWV glycoproteins is the Golgi complex where glycoproteins wrap around viral RNP complexes to form doubly enveloped virus particles (Kikkert et al., 1999). These virus particles are then fused with each other and with ER-derived membranes to form large intracellular vesicles that contain singly enveloped virus particles. Similarly, we assume that CaCV N protein aggregates may associate with viral RNA to form RNP complexes and traffic to the Golgi complex. In support we have shown N protein self-interaction and Gn-N interaction in the cytoplasm. Using fluorescence life-time imaging microscopy, Ribeiro et al. (2009) showed that TSWV Gc is retained in the ER, while Gn spread further to the Golgi when singly expressed in *N. tabacum* protoplasts. When these proteins were co-expressed, Gn was able to rescue Gc to co-migrate to the Golgi complex. Our transient expression experiments showed that CaCV Gn appears to partially localize at Golgi, whereas Gc was not, when expressed singly. When CaCV Gc and Gn were co-expressed there was no detectable alteration of localization profiles of these proteins. Non-Golgi localization of glycoproteins has been previously reported for INSV using the same Golgi marker (Dietzgen et al., 2012). In addition to cytoplasmic accumulation, both CaCV glycoproteins were also localized in the nucleus. Gn nuclear localization has so far not been reported for any other tospovirus and its function in the nucleus requires further investigation. Nuclear localization of Gc has been observed for INSV (Dietzgen et al., 2012) and IYSV (Tripathi et al., 2015a). Observed partial nuclear localization of CaCV glycoproteins is supported by predicted canonical NLSs. However, the biological significance of this nuclear localization cannot be explained based on current experimental data.

Tomato spotted wilt virus moves from cell-to-cell as a RNP complex (Kormelink et al., 1994) and NSm facilitates this movement through direct interaction with N protein (Soellick et al., 2000). Interactions of NSm and N proteins have also been reported for several tospoviruses other than TSWV (Dietzgen et al., 2012; Leastro et al., 2015; Singh and Savithri, 2015; Tripathi et al., 2015a). Therefore, we expected that CaCV NSm might interact with cognate N protein. However, BiFC assays did not show such an interaction in either orientation in repeated experiments, despite NSm being expressed and showing self-interaction. This may be due to steric hindrance or limited access to NSm N-terminus in the fusion protein as observed for some other tospoviruses previously studied (Leastro et al., 2015; Singh and Savithri, 2015). These studies showed NSm-N dimer formation of BeNMV, GBNV, and TSWV was restricted to certain combinations of fluorescent protein pairs tested. Therefore, potential interactions of CaCV NSm need to be confirmed using BiFC N-series vectors (viral protein N-terminus accessible due to fusion at C-terminus) and/or alternative protein–protein interaction assays. However, we cannot exclude other reasons for undetected interactions between CaCV NSm and N, such as involvement of host factors as bridges for viral protein interactions, like At-4/1 interactor of TSWV NSm (Paape et al., 2006). Association of cellular factors mediating connections between viral proteins that lead to cell-to-cell movement has also been described for plant rhabdoviruses (Min et al., 2010; Mann et al., 2016).

Recent studies have provided evidence for novel routes of intra- and intercellular trafficking of tospoviruses. Feng et al. (2013) showed cellular actin/ER membrane transport networks are involved in intracellular movement of TSWV N inclusions and ER network is involved in intercellular translocation of NSm and virus replication complexes where host factors are likely to play an important role (Feng et al., 2016). On the other hand, Singh and Savithri (2015) described vesicle-mediated intracellular transport of GBNV NSm. GBNV NSm directly interacts with ER membranes via a coiled-coil domain at the C-terminus, which remodels ER network to form vesicles that translocate NSm to PD. This property of GBNV NSm implies existence of potential alternate movement pathways for tospoviruses other than tubule formation. Furthermore, previous reports have shown that PD localization of NSm is apparently not a property shared by all tospoviruses. INSV and IYSV NSm did not have punctate distribution on the cell periphery suggesting these proteins do not form visible aggregates at PD (Dietzgen et al., 2012; Tripathi et al., 2015a). Taken together, localization and interaction profiles of tospovirus NSm proteins studied so far indicate potentially different strategies for intracellular trafficking, PD localization, and cell-to-cell movement (Paape et al., 2006; Dietzgen et al., 2012; Leastro et al., 2015, 2017; Singh and Savithri, 2015; Tripathi et al., 2015b; Feng et al., 2016). We speculate that different interacting host factors may be responsible for these differences.

We provide several lines of evidence to conclude that CaCV NSm represents the viral cell-to-cell movement protein. First, transiently expressed NSm localizes to and self-interacts on the cell periphery forming distinct punctate spots (**Figures 1**, **5**). Second, these NSm spots appear to coincide with PD because they perfectly co-localize with callose deposits stained with aniline blue (**Figure 4**). Third, NSm self-interacts at PD to form tubular structures that are predicted to traverse PD. Tubule-like structures composed of RFP:NSm were seen protruding from the periphery of protoplasts (**Figure 8**). Fourth, NSm functionally *trans*-complements a movement-defective tobamovirus replicon (**Figure 6**).

NSm was first identified as functional cell-to-cell movement protein of TSWV (Lewandowski and Adkins, 2005). Here we demonstrated that CaCV NSm was able to *trans*-complement movement of a heterologous tobamovirus replicon. The efficiency of movement complementation in this assay by CaCV NSm was 42% greater than that of TSWV NSm, but 22% less than the homologous TVCV P30. Further, the pattern of cell-to-cell movement of movement-defective TVCV replicon complement by CaCV NSm was similar to that of TVCV P30 where radial clusters of cells are formed, but different to TSWV NSm which led to lateral movement. These observations support the notion that differences may exist between tospoviruses in intercellular transport of virus replication complexes or virions. We also investigated whether D to A mutation in the analogous 30K-specific LxD/N_50-70_G motif in CaCV had an effect on cell-to-cell movement function and PD localization. Previously it was shown that TSWV D_154_ residue is essential for tubule formation and cell-to-cell movement (Li et al., 2009). Mutation of this residue in TSWV and CaCV NSm interfered with cell-to-cell movement activity of these proteins. In addition, CaCV NSm mutant no longer formed punctate spots on the cell periphery and was not localized at PD, suggesting CaCV D_155_ residue is essential for PD localization and cell-to-cell movement. Collectively our results show that CaCV NSm functions as a cell-to-cell MP that localizes at PD, can form tubules and facilitates cell-to-cell movement.

We have provided live cell imaging evidence for a unique localization and interaction profile of CaCV NSs in the cell periphery and in the nucleus when individually transiently overexpressed following agroinfiltration. Previous similar studies of INSV and IYSV NSs showed exclusive cytoplasmic localization and no interactions with other viral proteins (Dietzgen et al., 2012; Tripathi et al., 2015a). On the other hand, CaCV NSs self-interaction exclusively occurred in the nucleus. A predicted canonical NLS appeared to provide additional support for partially nuclear localization of CaCV NSs, but a similar NLS score was obtained for TSWV NSs, which was shown to localize to the cytoplasm (Kormelink et al., 1991; Kikkert et al., 1997). On the other hand, overexpressed INSV GFP-NSs did not accumulate in the nucleus and has no predicted NLS (Dietzgen et al., 2012). The nuclear homotypic CaCV NSs interaction occurred infrequently in only five nuclei of 20 observed, whereas >80% of nuclei accumulated NSs, suggesting that not all nuclear localized NSs proteins formed aggregates. Confocal microscopy of protoplasts expressing NSs detected by using a NSs MAb and immunofluorescence confirmed that >90% of observed nuclei accumulated NSs. To validate that NSs has a partial nuclear localization also during virus infection, transmission electron microscopy in combination with immunogold labeling of leaf thin sections from CaCV-infected plants should be done in future experiments. In the absence of a known nuclear export signal and partial accumulation in the cytoplasm, we speculate that NSs may shuttle between nucleus and cytoplasm through interacting with unknown host protein/s similar to the way that tombusvirus P19 partially translocates to the nucleus using ALY proteins (Canto et al., 2006). Furthermore, unlike other tospoviruses, CaCV NSs was uniquely shown to interact with N protein in the cytoplasm, an unexpected observation that warrants future study.

Bunyavirus NSs RNA silencing suppressor activity has only been clearly demonstrated for topoviruses, while for animal-infecting bunyaviruses it is still being debated (Hedil and Kormelink, 2016). CaCV NSs, like other tospoviral NSs proteins (Takeda et al., 2002; Schnettler et al., 2010; Goswami et al., 2012), functions as a suppressor of RNA silencing as evidenced by suppression of sense GFP-induced gene silencing both locally and systemically. Our local silencing suppression data show that GFP fluorescence in leaf patches expressing CaCV NSs remained bright at 7 dpi, whereas GFP expression was silenced in patches expressing TSWV NSs and negative control patches. RNA silencing induced locally may spread from initially silenced cells to adjacent cells through cell-to-cell movement of siRNA, which can be observed in leaves of *N. benthamiana* line 16c when exposed to UV light as a red halo surrounding the infiltrated patch (Himber et al., 2003). Wide red zones surrounding TSWV NSs and vector patches were visible at 7 dpi, while less pronounced red halo was observed surrounding the patch expressing CaCV NSs. This suggests that TSWV NSs does not appear to interfere with the short-distance spread of the silencing signal, while CaCV NSs appears to delay or interfere, but does not prevent the short-distance spread of the silencing signal. Examination of GFP-derived siRNAs may provide additional evidence for CaCV NSs effects on siRNA accumulation. Previously, NSs of TSWV, GRSV, PoLRSV, and TYRV were shown to suppress long-distance systemic RNA silencing (Hedil et al., 2015; Margaria et al., 2016). These authors suggest that NSs may interfere with biogenesis of the systemic silencing signal (secondary siRNAs). The same study showed that NSs mutants that lacked local silencing suppression activity were still able to suppress systemic silencing, suggesting that NSs may exclusively target a step essential for systemic silencing. In the case of CaCV NSs, it is unknown if there is a correlation between NSs nuclear localization and its suppression of silencing activity or viral pathogenicity, and this warrants future research.

Integrated localization and interaction data or interactomes of tospoviruses studied so far show both shared and distinct properties of viral proteins (**Figure 10**) that may ultimately facilitate virus multiplication and spread in the host plant. Furthermore, these data hint at the complex nature of viral-viral and probably viral-host protein interactions. In conclusion, the present study demonstrates that compared to other tospoviruses, CaCV proteins have both conserved and unique properties in terms of *in planta* localization, protein–protein interactions, and protein functions which likely effect viral multiplication and movement in host plants.

## Author Contributions

SWG and RD conceived and designed the experiments; SWG performed the experiments; SWG and RD analyzed the data; RD contributed reagents/materials/analysis tools; SWG and RD wrote the paper.

## Conflict of Interest Statement

The authors declare that the research was conducted in the absence of any commercial or financial relationships that could be construed as a potential conflict of interest.
