# Supplementary material for: Intracellular Localization, Interactions and Functions of Capsicum Chlorosis Virus Proteins
Source: Front Microbiol. 2017 Apr 11;8:612. doi: 10.3389/fmicb.2017.00612 (PMC5387057; doi:10.3389/fmicb.2017.00612)
Supplement: Supplementary file 1 [file Data_Sheet_1.DOCX]

***Supplementary Material***

**Intracellular localization, interactions and functions of capsicum chlorosis virus proteins**

**Shirani M.K. Widana Gamage and Ralf G. Dietzgen^*^**

**^*^Correspondence:** Ralf G. Dietzgen: [r.dietzgen@uq.edu.au](mailto:r.dietzgen@uq.edu.au)

**Supplementary Figures**

**
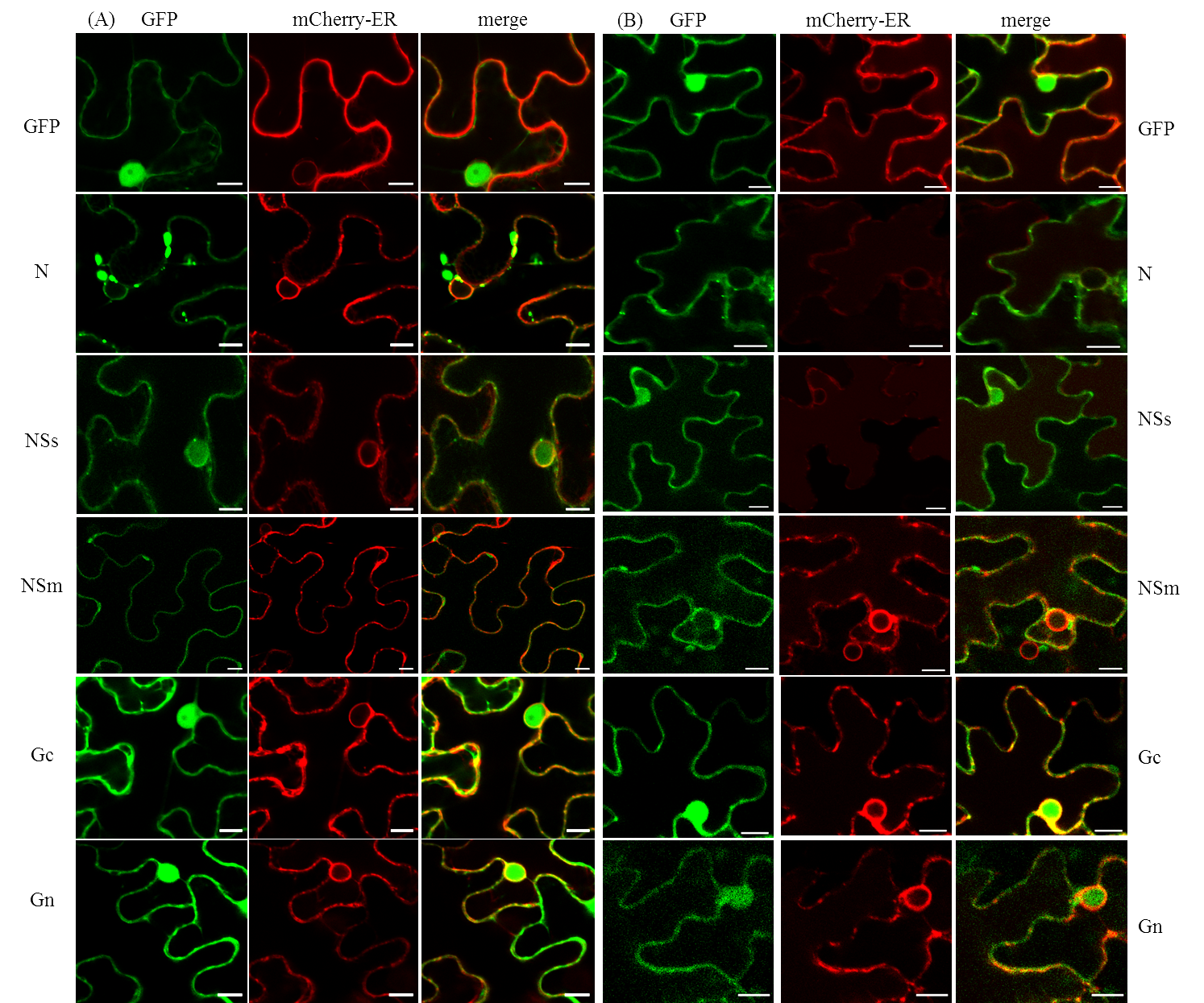
**

**Supplementary Figure 1.** Intracellular localization of transiently expressed free green fluorescent protein (GFP) or capsicum chlorosis virus (CaCV) proteins fused to GFP. CaCV proteins N, NSs, NSm, Gc, Gn were individually expressed from pSITE vectors, co-agroinfiltrated with mCherry-endoplasmic reticulum (ER) expression vector into (A) *Nicotiana benthamiana* or (B) capsicum leaf epidermal cells. Images were acquired after 2 days using a confocal microscope at 10 x 25 magnification. Left column, GFP channel; centre column, mCherry channel; right column, merged images. Bars, 10 µm.


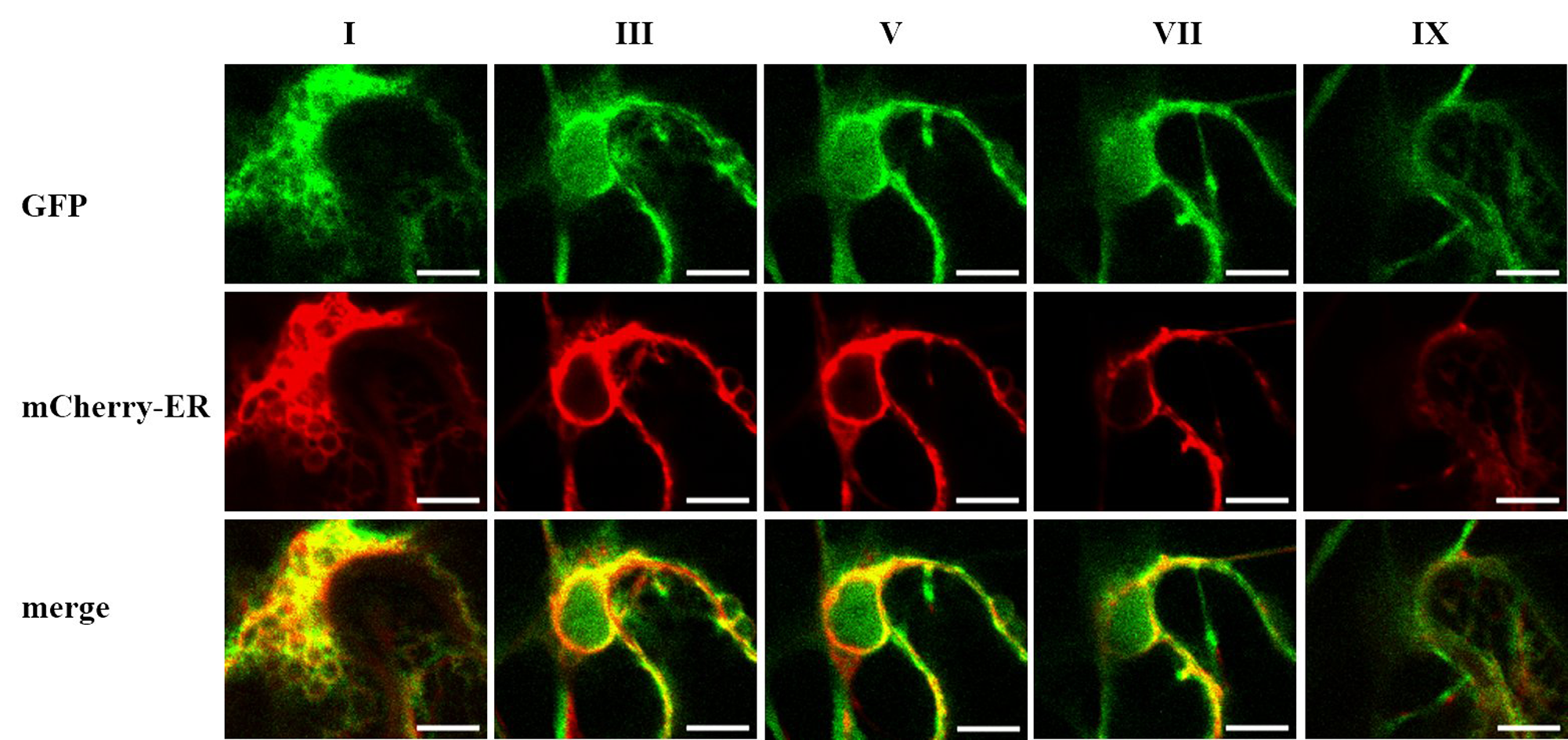


**Supplementary Figure 2.** ‘Z-stack’ confocal microscopy images showing nuclear and cytoplasmic localization of capsicum chlorosis virus NSs protein fused to green fluorescent protein (GFP) relative to mCherry-endoplasmic reticulum (ER) marker in virtual cross-sections. GFP:NSs expressed from pSITE-C1 vector was agroinfiltrated into *Nicotiana benthamiana* leaf epidermal cells and observed after 2 days. Shown are 5 images selected from 10 consecutive optical slices through the same group of cells. Optical slice number is shown above each column of images. Images were taken at 10 x 25 magnification. Bars, 10 µm.
